# Development and Validation of a Prognostic Nomogram for Prognosis in Patients With Renal Artery Stenosis

**DOI:** 10.3389/fmed.2022.783994

**Published:** 2022-04-11

**Authors:** Yan Li, Na Ma, Yuewei Zhang, Siyu Wang, Youjing Sun, Mengpu Li, Hu Ai, Hui Zhu, Yang Wang, Peng Li, Fajin Guo, Yongjun Li, Junhong Ren

**Affiliations:** ^1^Department of Sonography, Beijing Hospital, National Center of Gerontology, Institute of Geriatric Medicine, Chinese Academy of Medical Sciences, Beijing, China; ^2^Department of Cardiology, Beijing Hospital, National Center of Gerontology, Institute of Geriatric Medicine, Chinese Academy of Medical Sciences, Beijing, China; ^3^Department of Nuclear Medicine, Beijing Hospital, National Center of Gerontology, Institute of Geriatric Medicine, Chinese Academy of Medical Sciences, Beijing, China; ^4^Department of Medical Research & Biometrics Center, National Center for Cardiovascular Diseases and Fuwai Hospital, Chinese Academy of Medical Sciences and Peking Union Medical College, Beijing, China; ^5^Beijing Institute of Geriatrics, National Center of Gerontology, Institute of Geriatric Medicine, Chinese Academy of Medical Sciences, Beijing, China; ^6^Department of Vascular Surgery, Beijing Hospital, National Center of Gerontology, Institute of Geriatric Medicine, Chinese Academy of Medical Sciences, Beijing, China

**Keywords:** renal artery stenosis, prognosis prediction, renal cortical blood perfusion, scoring system, contrast-enhanced ultrasound

## Abstract

**Background and Objective:**

Renal artery stenosis (RAS) is associated with an increased risk of renal function deterioration (RFD). Our previous study showed that renal cortical blood perfusion assessed by contrast-enhanced ultrasound (CEUS) was an important related factor for RFD in RAS patients. Based on several conventional related factors confirmed by previous studies, we aimed to establish and verify a CEUS+ scoring system to evaluate the risk of RFD at 1 year of follow-up in RAS patients.

**Methods:**

This study was a single-center retrospective study. A total of 497 elderly RAS patients (247 in the training group and 250 in the verification group) admitted to the Beijing Hospital from January 2016 to December 2019 were included. The baseline characteristics of the patients on admission (including general conditions, previous medical history, blood pressure, blood creatinine, RAS, and cortical blood perfusion in the affected kidney) and renal function [glomerular filtration rate (GFR)] at 1-year of follow-up were collected. We used the univariate and multivariate logistic regressions to establish a CEUS+ scoring system model, the receiver operating characteristic (ROC) curve and area under the curve (AUC) to evaluate prediction accuracy, and the decision curve analysis and nomogram to evaluate the clinical application value of CEUS+ scoring system model.

**Results:**

Among the 497 patients enrolled, 266 (53.5%) were men, with an average age of (51.7 ± 19.3) years. The baseline clinical-radiomic data of the training group and the verification group were similar (all *p* > 0.05). Multivariate logistic regression analysis results showed that age [Odds ratio (OR) = 1.937, 95% confidence interval (CI): 1.104–3.397), diabetes (OR = 1.402, 95% CI: 1.015–1.938), blood pressure (OR = 1.575, 95% CI: 1.138–2.182), RAS (OR = 1.771, 95% CI: 1.114–2.816), and area under ascending curve (AUCi) (OR = 2.131, 95% CI: 1.263–3.596) were related factors for the renal function deterioration after 1 year of follow-up (all *p* < 0.05). The AUC of the ROC curve of the CEUS+ scoring system model of the training group was 0.801, and the Youden index was 0.725 (specificity 0.768, sensitivity 0.813); the AUC of the ROC curve of the validation group was 0.853, Youden index was 0.718 (specificity 0.693, sensitivity 0.835). There was no significant difference in ROC curves between the two groups (*D* = 1.338, *p* = 0.325). In addition, the calibration charts of the training and verification groups showed that the calibration curve of the CEUS+ scoring system was close to the standard curve (*p* = 0.701, *p* = 0.823, both *p* > 0.10).

**Conclusion:**

The CEUS+ scoring system model is helpful in predicting the risk of worsening renal function in elderly RAS patients.

## Introduction

Renal artery stenosis (RAS) mainly refers to the narrowing of the main artery or branch of the renal artery, which leads to renal ischemia, and the activity of the renin-angiotensin system is significantly increased, resulting in hypertension and renal dysfunction (1, 2). When RAS is severe to a certain degree, it can cause renal artery hemodynamic abnormalities, resulting in renal blood flow perfusion and other changes in renal function. Therefore, some patients with mild to moderate RAS can show renal function decline (3). Vessel reconstruction and drug therapy are important methods for the treatment of moderate to severe RAS. However, large-scale randomized controlled clinical studies such as ASTRAL and CORAL have showed that stenting combined with drug therapy did not improve the prognosis of patients with moderate to severe RAS (lumen stenosis ≥ 60%) (4, 5). Therefore, it is warranted to find the related factors for prognosis, especially the risk of renal function deterioration (RFD), which account for more than half of all the adverse cardiac and renovascular events (4, 5).

Previous studies have mostly investigated the prognosis of RAS patients in terms of clinical characteristics. Several studies have confirmed that age, diabetes, and severe hypertension were conventional factors for RFD in RAS patients (6). In addition, for patients with severe stenosis requiring stenting, the degree of RAS was not significantly related to the risk of RFD (7, 8). However, with the progress of contrast-enhanced ultrasound (CEUS) and other imaging omics, renal parenchymal blood perfusion was also an important factor (9). We have found that CEUS can evaluate renal parenchymal blood perfusion in real time, quantitatively and in a safe manner. Furthermore, cortical blood perfusion in patients with mild, moderate, and severe RAS was significantly different, and cortical blood perfusion parameters were significantly related to RFD detected by radionuclide renal imaging (10, 11). Therefore, based on conventional factors for RFD, we aimed to compare and analyze the relationship between renal cortical blood perfusion and RFD, and establish and validate a CEUS+ scoring system for predicting the risk of RFD at 1 year of follow-up.

## Subjects and Methods

### Subjects

This was a single-center retrospective study. A total of 497 elderly RAS patients admitted to the Beijing Hospital from January 2016 to December 2019 were included. Among the 497 patients, 266 (53.5%) were men with an average age of (51.7 ± 19.3) years. Patients were assigned to the training group (247 cases) and the verification group (250 cases) in a 1:1 ratio.

Inclusion criteria: (1) clinically diagnosed RAS patients (diagnosed by digital subtraction angiography (DSA), CT angiography (CTA), or CEUS; the lumen diameter is reduced by 30% to 99%) (12); (2) aged 18 to 85 years, regardless of gender; (3) underwent CEUS examination to assess renal cortical blood perfusion; (4) having complete follow-up data.

Exclusion criteria: (1) primary hypertension; (2) combined with severe cardiopulmonary dysfunction; (3) hypersensitivity to the contrast agent sulfur hexafluoride; (4) no stenosis of the renal artery (decrease in lumen diameter < 30%) or renal artery occlusion (100% reduction in lumen diameter); (5) ultrasound and other imaging images were not clear; (6) with advanced tumors; (7) pregnant women; (8) did not cooperate to treatment; (9) refused to sign informed consent. This study has been registered in the China Clinical Trial Registration Center (ChiCTR1800016252), meets medical ethics requirements, and has been reviewed and approved by the ethics committee of our hospital (2018BJYYEC-043-02).

### Methods

Patients’ baseline characteristics, such as demographic data, clinical data, and biochemical examinations, as well as imaging data (degree of RAS, cortical blood perfusion detected by CEUS) and follow-up data (renal function at 1 year of follow-up) were recorded.

### Renal Cortical Blood Flow Perfusion

Contrast-enhanced ultrasound examination was performed on all patients with a Samsung ultrasound system to evaluate renal cortical blood flow perfusion. The starting imaging conditions were as follows: mechanical index 0.12, image depth 15 cm, and gain 30 dB. The patient was injected twice with the contrast agent on each side of the kidney, and the main renal artery (dose 1.0 ml/kidney) and renal cortical perfusion (dose 1.2 ml/kidney) were observed. First, the patient was made to lie on the side, and the long-axis section of the kidney was fixed so that it was perpendicular to the direction of the sound beam. The contrast mode was opened. The contrast agent was then injected through the cubital vein, and the renal cortex contrast agent perfusion storage image was dynamically observed for 3 min. Renal cortex blood perfusion parameters were analyzed, including area under ascending curve (AUCi), area under the descending curve (AUCo), peak intensity (PI), time to peak intensity (TTP), and mean transit time (MTT) (12).

### Renal Glomerular Filtration Rate

All patients underwent radionuclide renal imaging to evaluate the glomerular filtration rate (GFR) of the kidney, with Symbia E-type SPECT or Symbia T16-type SPECT/CT (Siemens, Germany) under a low-energy high-resolution collimator. Images were collected by GATES method, and the radioactivity counts of the full and empty needles were measured at 30 cm before the probe, before and after injection for 6 s. The patient was placed in a supine position, and the probe field included both kidneys. After injection of 99mTc-DTPA (Atom High-Tech Co. Ltd.), 1.85 Bq × 108 Bq, through the cubital vein, the computer dynamic collection was started immediately. The operation process was divided into two groups and images acquisition matrix was 64 × 64. In the first group of blood perfusion phase: 2 s/frame, 30 frames were collected. In the second group of intake and excretion phase: 60 s/frame, 20 frames were collected, for a total of 20 min. Using region of interest (ROI) technology, the images were processed to draw the blood flow perfusion, uptake, and excretion curves of the bilateral kidneys. The GATES method was used to determine the total GFR and sub-renal GFR (ml/min).

### Deterioration of Renal Function

Deterioration of renal function refers to the estimated GFR (eGFR), which is reduced by ≥ 30% after treatment compared with before treatment, and lasts for at least 60 days, while the deterioration of renal function caused by other reasons is excluded (4, 5).

### Statistical Method

Stata 16.0 statistical software was used, and normal distribution measurement data was expressed by mean ± standard deviation. Comparison between the groups was performed by independent sample *t*-test or continuous correction *t*-test. Count data was expressed as percentage composition ratio, and comparison of rate between the groups was by χ^2^ test.

Univariate and multivariate logistic regressions were used to evaluate the related factors for renal function deterioration and to establish the CEUS+ scoring system model. The receiver operating characteristic (ROC) curve and the area under the curve (AUC) were used to evaluate the prediction accuracy. The decision curve analysis and nomogram were used to evaluate the CEUS+ scoring system model. Clinical application value of *p* < 0.05 was considered statistically significant.

## Results

### Baseline Characteristics

There were no significant differences in the general condition, previous medical history, blood pressure, blood creatinine, degree of RAS, and cortical blood flow perfusion in the training group and the verification group (all *p* > 0.05) (Table 1).

**TABLE 1:** Baseline characteristics of the training group and verification group.

Data	Training group (*n* = 247)	Verification group (*n* = 250)	*P*-value
**Demographic data**			
Age (year)	52.2 ± 27.3	51.3 ± 28.5	>0.05
Male [n (%)]	137 (55.5)	129 (51.6)	>0.05
Hypertension duration (year)	14.2 ± 10.4	13.3 ± 9.7	>0.05
**Previous history[n (%)]**			
Diabetes	88 (35.6)	92 (36.8)	>0.05
Hyperlipidemia	97 (39.3)	104 (41.6)	>0.05
Smoking	145 (58.7)	152 (60.8)	>0.05
Coronary artery disease	66 (26.7)	73 (29.2)	>0.05
**Hypertension (mmHg)**			
Systolic blood pressure	153.9 ± 33.8	156.2 ± 31.7	>0.05
Diastolic blood pressure	99.2 ± 15.9	98.9 ± 19.2	>0.05
Mean average pressure	115.6 ± 30.2	117.7 ± 29.3	>0.05
**Lab. test**			
Serum creatinine (μmol/L)	78.3 ± 20.5	80.6 ± 18.7	>0.05
**Imaging**			
Lumen stenotic degree (%)	65.9 ± 27.8	66.5 ± 30.2	>0.05
RAS classification [n (%)]			>0.05
Mild stenosis	45 (18.2)	41 (16.4)	
Moderate stenosis	122 (49.4)	133 (53.2)	
Severe stenosis	80 (32.4)	76 (30.4)	
**Renal cortical blood perfusion**			
AUCi (dB × s)	1127.3 ± 672.6	1133.5 ± 696.8	>0.05
AUCo (dB × s)	47612.2 ± 1892.6	4690.7 ± 1916.2	>0.05
PI (dB)	120.23 ± 32.1	121.1 ± 30.7	>0.05
TTP (s)	18.2 ± 9.4	19.5 ± 9.6	>0.05
MTT (s)	48.7 ± 16.0	47.1 ± 18.5	>0.05
GFR (ml/min)	72.5 ± 34.8	74.1 ± 37.2	>0.05

### Univariate and Multivariate Logistic Regression Analyses

Univariate logistic regression analysis showed that age, diabetes, systolic blood pressure, diastolic blood pressure, blood creatinine, GFR, AUCi, AUCo, PI, and MTT were risk factors for function deterioration (all *p* < 0.05).

Multivariate logistic regression analysis found that age (OR = 1.937, 95% CI: 1.104–3.397), diabetes (OR = 1.402, 95% CI: 1.015–1.938), blood pressure (OR = 1.575, 95% CI: 1.138–2.182), RAS (OR = 1.771, 95% CI: 1.114–2.816), and AUCi (OR = 2.131, 95% CI: 1.263–3.596) were related factors for the renal function deterioration after 1 year of follow-up (all *p* < 0.05) (Table 2).

**TABLE 2:** Multivariate logistic regression analysis.

Risk factor	β value	SE value	Wald χ^2^ value	OR	95%CI	*P*-value
Age	0.661	0.287	0.523	1.937	1.104–3.397	0.022
Diabetes	0.338	0.162	0.705	1.402	1.015–1.938	0.041
Hypertension	0.454	0.166	1.243	1.575	1.138–2.182	0.006
RAS	0.572	0.222	1.472	1.771	1.114–2.816	0.014
AUCi	0.757	0.268	2.136	2.131	1.263–3.596	0.005

### Establishment of CEUS+ Scoring System Model

**FIGURE 1:**
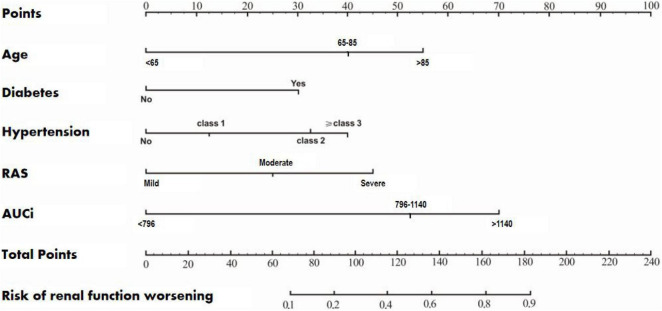
A nomogram of the CEUS+ scoring system for predicting the rate of deterioration of renal function during the 1-year follow-up.

According to the results of multivariate logistic regression analysis, a prognostic prediction model, i.e., a nomogram of the CEUS+ scoring system, was drawn, and its C-Index was 0.733 (95% CI: 0.653–0.822) (Figure 1). We used the Bootstrap method to perform re-sampling for 1000 times. The sample size was 147 and was used to verify the predictive value of CEUS+ scoring system nomogram for the risk of renal function deterioration. The correlation of the rate of renal function deterioration at 1 year of follow-up was good, which indicates that the prediction accuracy of the nomogram was high.

### Validation of CEUS+ Scoring System Models

**FIGURE 2:**
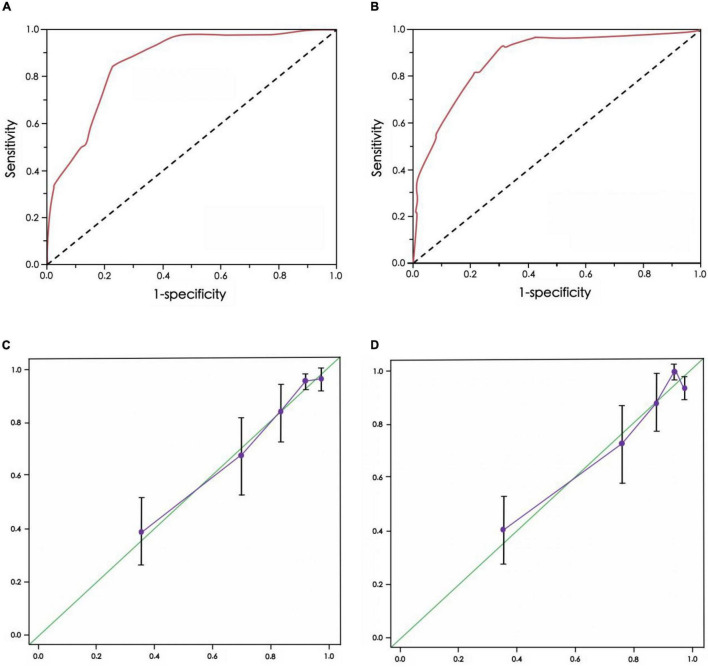
Receiver operating characteristic (ROC) curve and calibration chart of CEUS+ scoring system model for predicting the risk of renal function deterioration in patients. **(A)** ROC curve of training group, **(B)** ROC curve of verification group, **(C)** Calibration chart of training group, **(D)** Calibration chart of verification group

The AUC of the ROC curve of the CEUS+ scoring system model of the training group was 0.801, and the Youden index was 0.725 (specificity 0.768, sensitivity 0.813) (Figure 2A). The AUC of the ROC curve of the validation group was 0.853, and the Youden index was 0.718 (specificity 0.693, sensitivity 0.835) (Figure 2B). There was no significant difference in ROC curves between the two groups (*D* = 1.338, *p* = 0.325).

In addition, the calibration charts of the training group and the verification group showed that the calibration curve of the CEUS+ scoring system model was close to the standard curve, and the difference was not statistically significant (*p* = 0.701, *p* = 0.823, both *p* > 0.10) (Figures 2C,D).

## Discussion

This study established and validated a CEUS+ scoring system for predicting the risk of RFD in RAS patients who were followed for 1 year. The model consists of clinical risk factors (age, diabetes, hypertension, and RAS) and CEUS imaging features (AUCi). The visualization of the model was achieved through the application of nomograms, and the clinical application value of the CEUS+ scoring system model was confirmed through verification. Therefore, the CEUS+ scoring system can help predict the short-term risk of RFD, thereby helping to stratify patients (13).

Various clinical risk factors can affect the prognosis of RAS patients. A number of previous studies have confirmed that advanced age, diabetes, and severe hypertension were important factors for prognosis in RAS patients, but the degree of RAS was not a related factor (14, 15). Similar to the previous study, in a recent study involving 400 patients with peripheral arterial ischemia, DSA confirmed 57 patients with RAS. After an average follow-up of 62 months, it was also found that RAS was not a related factor for the primary outcome (all-cause death, peripheral vascular reperfusion treatment, and amputation) and secondary outcome (all-cause death, MI, stroke, and coronary or carotid reperfusion therapy), while age, insulin application, and severe limb ischemia were risk factors for all-cause mortality (16). Similar to previous studies, our study found that age, diabetes, and hypertension were all risk factors for RFD. Meanwhile, we also found that the degree of RAS was significantly related to the risk of RFD. For RAS patients who required stenting for moderate to severe stenosis, CORAL subgroup analysis showed that the severity of hypertension and the difference in peak systolic flow velocity at both ends of the stenotic lesion were risk factors that affected the prognosis of stenting, and patients with a low urinary albumin/creatinine level (<22.5 mg/g) had a tendency of better prognosis (17). However, unlike the CORAL study, the patients included in our study were younger patients, with an average age of (51.7 ± 19.3) years, with a decreased level of artery stenosis (66.2 ± 24.5)%. Meanwhile, the follow-up time was shorter, and risk of RFD was the primary outcome. So, it may affect the judgment of risk factors, and the follow-up time needs to be gradually increased to guide clinical practice more accurately.

Renal cortical blood perfusion was an important imaging index that affected the prognosis of RAS patients. Feng Qichen et al. (7) found that the renal filtration fraction increased significantly before stenting by using 99mTc-EC to measure the effective renal plasma flow, combined with radionuclide renal imaging to determine GFR, and calculate the renal filtration fraction (normally 18–22%). Patients with normal preoperative renal filtration scores had partially improved renal function, while those with a reduced preoperative renal filtration score had a poor prognosis. Chrysochou et al. (8) found that patients with high scores of renal GFR, by using magnetic resonance measurement of renal parenchymal blood flow and nuclide-detected GFR ratio of kidneys, were associated with significantly improved renal function after stent treatment. In this study, we found that CEUS-detected cortical blood perfusion was also an influential factor in the prognosis of RAS patients. AUCi was one of the independent risk factors for RFD during 1 year of follow-up. Therefore, renal cortical blood perfusion is a useful supplement for clinicians to evaluate the degree of renal ischemia, judge the prognosis, and guide the treatment of RAS revascularization (18).

At present, although many studies have found that age, diabetes, and blood pressure are risk factors affecting the prognosis of elderly RAS patients (14–17), no previous study had established a model to predict the risk of RFD in RAS patients (19). Based on a total of five risk factors such as age, diabetes, hypertension, RAS, and AUCi, we established a nomogram of the CEUS+ scoring system. The C-Index was 0.733 (95% CI: 0.653–0.822), suggesting that the CEUS+ scoring system’s nomogram prediction was accurate, with high clinical application value (20, 21).

### Limitations

This study has some limitations, which are listed below. (1) This study was a single-center study, and more centers were needed to verify the versatility of this study. (2) We changed the continuous variables to categorical variables, which may affect the accuracy of the model. (3) We used the cortical blood perfusion parameters assessed on admission, not the changes of cortical blood perfusion before and after treatment, which may affect the predictive accuracy of the model. (4) We used the logistic regression analyses method. However, logistic regression analyses had multicollinearity problem and the occurrence of renal function deterioration depended on the follow-up duration. LASSO regression and Cox regression methods could reduce these risk. (5) The follow-up time of this study was short, and the risk of RFD was used as the primary outcome indicator. Adopting the hard index of adverse cardiovascular and renal events will also reduce the clinical value of this model. Therefore, we need to further optimize the prediction model in subsequent research to improve the accuracy of prediction (22–26).

## Conclusion

In summary, clinical-imaging features such as age, diabetes, blood pressure, RAS, and AUCi were risk factors for RFD at 1 year of follow-up. The CEUS+ scoring system established on this basis can accurately predict the prognosis, and thus can be used to stratify patients and guide treatment. Therefore, the CEUS+ scoring system has high clinical application value, but more studies are needed to confirm it.

## Data Availability Statement

The raw data supporting the conclusions of this article will be made available from the corresponding author by request.

## Ethics Statement

The studies involving human participants were reviewed and approved by the Beijing Hospital. The patients/participants provided their written informed consent to participate in this study.

## Author Contributions

YaL: data collection and analyses. JR: study supervision. All authors contributed to the article and approved the submitted version.

## Conflict of Interest

The authors declare that the research was conducted in the absence of any commercial or financial relationships that could be construed as a potential conflict of interest.

## Publisher’s Note

All claims expressed in this article are solely those of the authors and do not necessarily represent those of their affiliated organizations, or those of the publisher, the editors and the reviewers. Any product that may be evaluated in this article, or claim that may be made by its manufacturer, is not guaranteed or endorsed by the publisher.
